# Supplementary material for: Superior colliculus bidirectionally modulates choice activity in frontal cortex
Source: Nat Commun. 2023 Nov 14;14:7358. doi: 10.1038/s41467-023-43252-9 (PMC10645979; doi:10.1038/s41467-023-43252-9)
Supplement: Supplementary file 1 — Supplementary Information [file 41467_2023_43252_MOESM1_ESM.pdf]

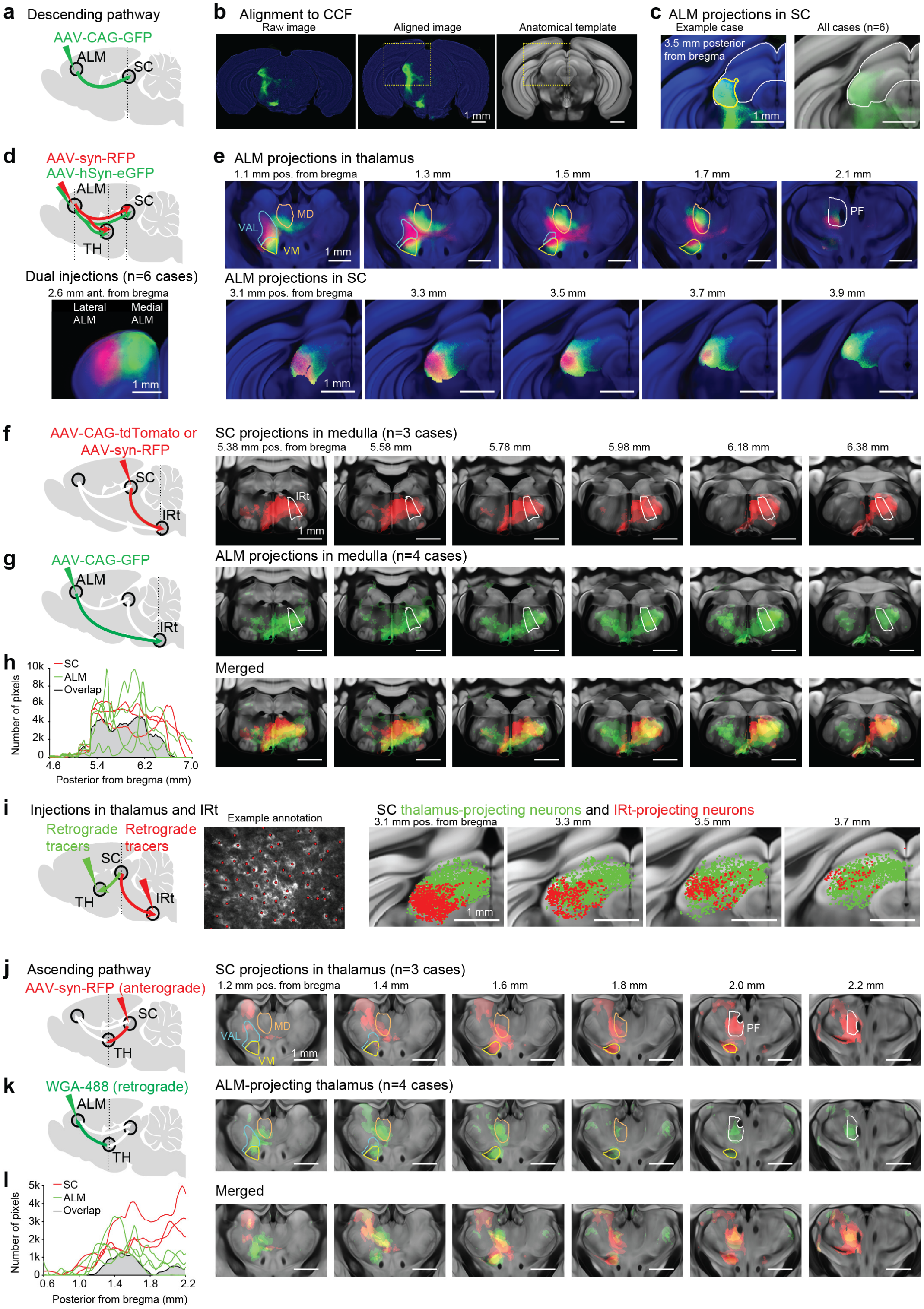

Supplementary Fig. 1 | Descending and ascending anatomical pathways of the ALM-SC circuit.

- **a.** Anterograde tracer injections in the left ALM to map its descending projections in SC.
- **b.** Alignment of anatomical data into the Allen Mouse Common Coordinate Framework (CCF). *Left*, an example coronal section containing SC. Fluorescence shows anterograde labeling from ALM. *Middle*, the coronal section after alignment to the CCF. *Right*, corresponding coronal section of the CCF anatomical template. Yellow dashed line, a region of interest containing SC.

c. *Left*, the highlighted region of interest from **b** showing ALM projections in SC. The aligned image containing fluorescence labeling (green) is overlaid onto the CCF anatomical template (blue). White line, SC border from the Allen Reference Atlas. Yellow outline, labeled area in SC after thresholding of fluorescence (Methods). *Right*, average fluorescence intensity (green) from all injection cases (n=6).

d. Two-colored anterograde tracer injections in the medial and lateral ALM to map the organization of ALM descending projections. *Top*, injection schematic. In different brains, green and red fluorescent proteins were used for either the medial ALM or lateral ALM (Methods). For display, colors are inverted so that the medial ALM injections are always in green. *Bottom*, coronal section showing the average fluorescence from all injection cases (n=6 cases).

e. Topographically organized ALM projections in the thalamus and SC. *Top*, average fluorescence intensity in the thalamus from all injection cases (n=6). Borders of thalamic nuclei: VAL, ventral anterior-lateral complex; VM, ventral medial nucleus; MD, mediodorsal nucleus; PF, parafascicular nucleus. *Bottom*, average fluorescence intensity in SC. Fluorescence outside of SC is excluded for visualization purposes. Green, anterograde labeling from the medial ALM; red, anterograde labeling from the lateral ALM.

f. *Left*, anterograde tracer injections in the lateral region of left SC to map its descending projections in the medulla. *Right*, anterograde labeling from the lateral SC (n=3 cases). The labeled areas (red) are based on thresholding of fluorescence (Methods, see example in **c**). IRt, intermediate nucleus of the reticular formation. White arrows show unlabeled parts of the descending pathway.

g. *Left*, anterograde tracer injections in the left ALM to map its descending projections in the medulla. *Right*, same as **f** but for ALM injections (green, n=4 cases).

h. *Left*, labeled area in the medulla by left SC injections (red, n=3) and left ALM injections (green, n=4). Black, the average size of the co-labelled area. *Right*, merged view of ALM and SC projections in the medulla.

i. Organization of SC thalamus-projecting neurons and IRt-projecting neurons. *Left*, two-colored retrograde tracer injections in the thalamus and IRt and an example image showing labeled neurons in SC from an IRt injection. Labeled neurons are manually annotated (red dots, Methods). *Right*, organization of SC thalamus-projecting neurons (green) and IRt-projecting neurons (red). Annotated neurons from 3 cases of thalamus injections and 3 cases of IRt injections are shown in CCF.

j. *Left*, anterograde injections in the lateral region of left SC to map its ascending projections in the thalamus. *Right*, anterograde labeling from the lateral SC (n=3 cases). The labeled area (red) is based on thresholding of fluorescence (Methods, see example in **c**). White arrows show unlabeled parts of the cortico-collicular loop.

k. *Left*, retrograde injections in the left ALM to label the ALM-projecting thalamus. *Right*, same as **j** but for ALM injections (green, n=4 cases).

l. *Left*, labeled area in the thalamus by left SC injections (red, n=3) and left ALM injections (green, n=4). 3 cases contain co-injections in ALM and SC. Black, the average size of the co-labelled area. *Right*, merged view of SC projections and ALM-projecting thalamus.

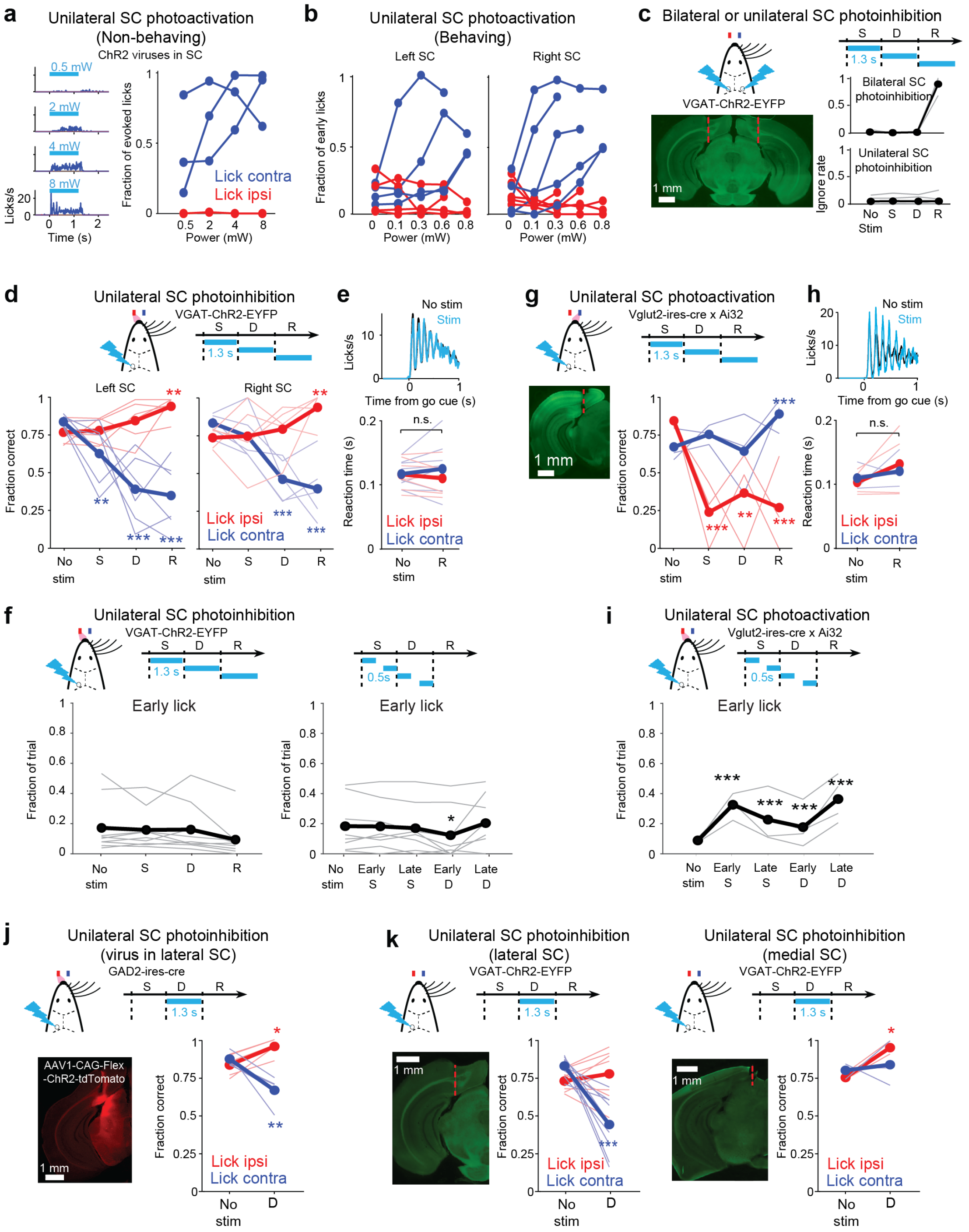

Supplementary Fig. 2 | Effects of SC manipulations on behavior.

- **a.** Unilateral SC photoactivation in non-behaving mice triggers contralateral licking across a range of powers. Non-behaving mice are trained in the task, but the mice were tested outside of the task in absence of sensory stimulus and reward. *Left,* example coronal section with cannula labelled in the lateral SC (red line). *Middle,* average lick rate during photostimulation (cyan) from an example mouse. *Right,* fraction of trials in which photostimulation causes licking as a function of laser power. Lick responses are broken out by the direction of licking relative to the manipulated hemisphere (blue, contralateral licking; red, ipsilateral licking). Individual lines, individual mice (n=3).
- **b.** Unilateral photoactivation of left or right SC triggers contralateral licking during behavior across a range of powers. Fraction of trials in which photostimulation during the sample or delay epoch causes an ‘early lick’ as a function of laser power. Individual lines, individual mice (left SC photostimulation, n=4; right SC photostimulation, n=5).

c. Bilateral, but not unilateral, photoinhibition of SC during the response epoch blocks licking. *Left,* example coronal section with cannula labelled in the lateral SC (red line). *Right,* fraction of trials with no lick response (ignore rate) as a function of stimulation condition. Black line, mean; gray lines, individual mice (n=4 for bilateral photoinhibition, 7 for unilateral photoinhibition).

d. Unilateral SC photoinhibition during the sample, delay, or response epoch produces ipsilateral biases in lick direction. Thick lines, mean; thin lines, individual mice (left SC photoinhibition, n=6; right SC, n=5). **p<0.01; ***p<0.001; one-tailed test, bootstrap (Methods). "Lick left" and "lick right" trials are grouped by instructed lick direction relative to the manipulated hemisphere. Blue, contralateral (lick contra); red, ipsilateral (lick ipsi). Photostimulation power, 1.2 mW.

e. Unilateral SC photoinhibition does not significantly alter licking execution and reaction time. Photostimulation data is from response epoch photoinhibition. *Top,* average lick rate in photostimulation (cyan) versus control trials (black). *Bottom,* reaction time as a function of stimulation condition. Reaction time is from the onset of the go cue to the first contact with the lickport. n.s., p>0.05, one-tailed test, bootstrap (Methods).

f. Unilateral SC photoinhibition does not evoke significantly more early licking. Thick lines, mean; thin lines, individual mice. *Left*, photoinhibition during the sample, delay, or response epoch (n=9). *Right*, photoinhibition during sub-epochs of the sample or delay epoch (n=8). * p<0.05, one-tailed test, bootstrap (Methods).

g. Unilateral SC photoactivation produces contralateral biases in lick direction. Same as **d** but for SC photoactivation (n=4). Photostimulation power, 0.1-0.2 mW.

h. Unilateral SC photoactivation does not significantly alter licking execution and reaction time. Same as **e** but for SC photoactivation.

i. SC photoactivation slightly increases early lick rate. Same as **f** but for SC photoactivation. *** p<0.001, one-tailed test, bootstrap (Methods).

j. Alternative SC photoinhibition strategy also biases lick direction. ChR2 was expressed in SC GABA neurons using AAV1-CAGGS-Flex-ChR2-tdTomato virus injections into GAD2-ires-cre mice. *Left,* example coronal section with fluorescence (red) indicating SC virus expression. *Right*, behavioral performance as a function of stimulation condition. N=4 mice. *p<0.05; **p<0.01; one-tailed test, bootstrap (Methods).

k. Medial SC photoinhibition does not induce ipsilateral biases in lick direction. *Left,* photoinhibition of the lateral SC. Example coronal section shows cannula placement in the lateral SC (red line). Data from **d**, delay epoch photostimulation. N=9 mice. *Right*, photoinhibition of the medial SC. N=3 mice. *p<0.05, ***p<0.01; one-tailed test, bootstrap (Methods).

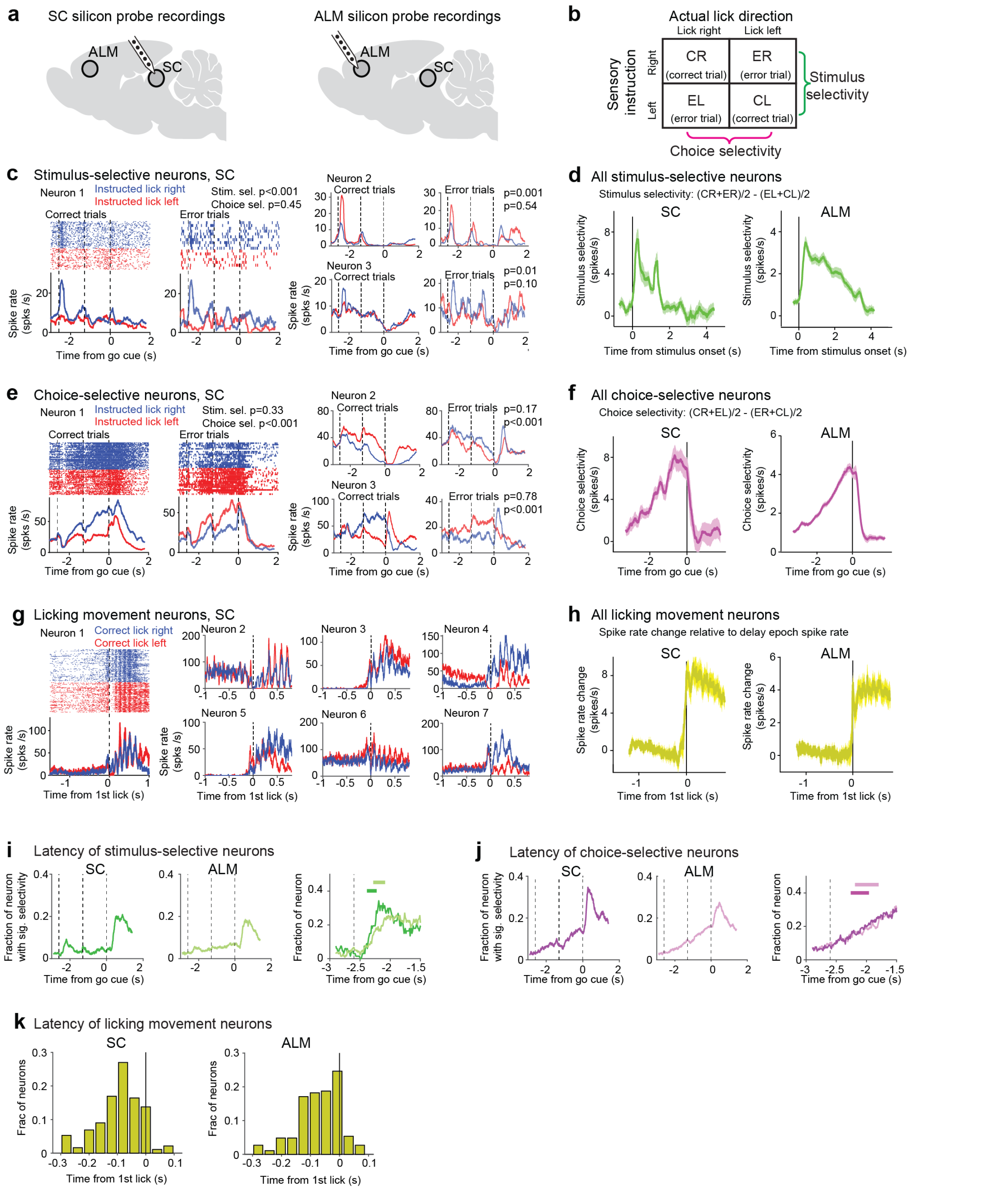

Supplementary Fig. 3 | ALM and SC neurons show stimulus, choice, and movement activity.

- **a.** Silicon probe recordings in ALM and SC.
- **b.** Calculating stimulus and choice selectivity using correct and error trials. Significant selectivity is tested with three-way ANOVA (Methods)
- **c.** 3 example stimulus-selective neurons in SC. Peristimulus time histograms (PSTHs) are shown for correct and error trials. Trial types are colored based on sensory instruction (blue, “lick right”; red, “lick left”). The same trial-type preference in correct and error trials indicates selectivity for object location (stimulus).

d. Population selectivity for stimulus-selective neurons in SC (n=41) and ALM (n=336). Stimulus selectivity is the difference in spike rate between the preferred and non-preferred trial type defined by object location (correct and error trials combined, Methods). Mean ± s.e.m. across neurons.

e. 3 example choice-selective neurons in SC. The reversed trial-type preference in correct and error trials indicates selectivity for lick direction (choice).

f. Population selectivity for choice-selective neurons in SC (n=70) and ALM (n=464). Choice selectivity is the difference in spike rate between the preferred and non-preferred trial type defined by lick direction (correct and error trials combined, Methods).

g. 7 example licking movement neurons in SC. Licking movement neurons are defined as neurons with significant spike rate modulation during rhythmic licking cycles (Methods). Spike times are aligned to the first lick. PSTHs are shown for correct “lick right” (blue) and “lick left” trials (red).

h. Population activity for licking movement neurons in SC (n=264) and ALM (n=208). Activity shows the difference in spike rate between the response epoch and delay epoch (averaged across trial types).

i. Stimulus selectivity emerges simultaneously in SC and ALM. *Left*, fraction of neurons in SC and ALM with significant stimulus selectivity. Fraction is relative to all neurons in each region. *Right*, emergence of stimulus-selective neurons during the sample epoch. Fraction is relative to all neurons with significant selectivity during sample or delay epoch. Bars on top show latency defined as when the fraction of selective neurons reaches 50% of its peak.

j. Choice selectivity emerges simultaneous in SC and ALM. Same as **i** but for choice-selective neurons.

k. Licking movement neurons are typically active before the first lick, consist with a motor command that drives licking. Latency is the first time bin (20 ms) in which activity deviates significantly from the delay epoch spike rate ($p<0.01$, two-tailed t-test).

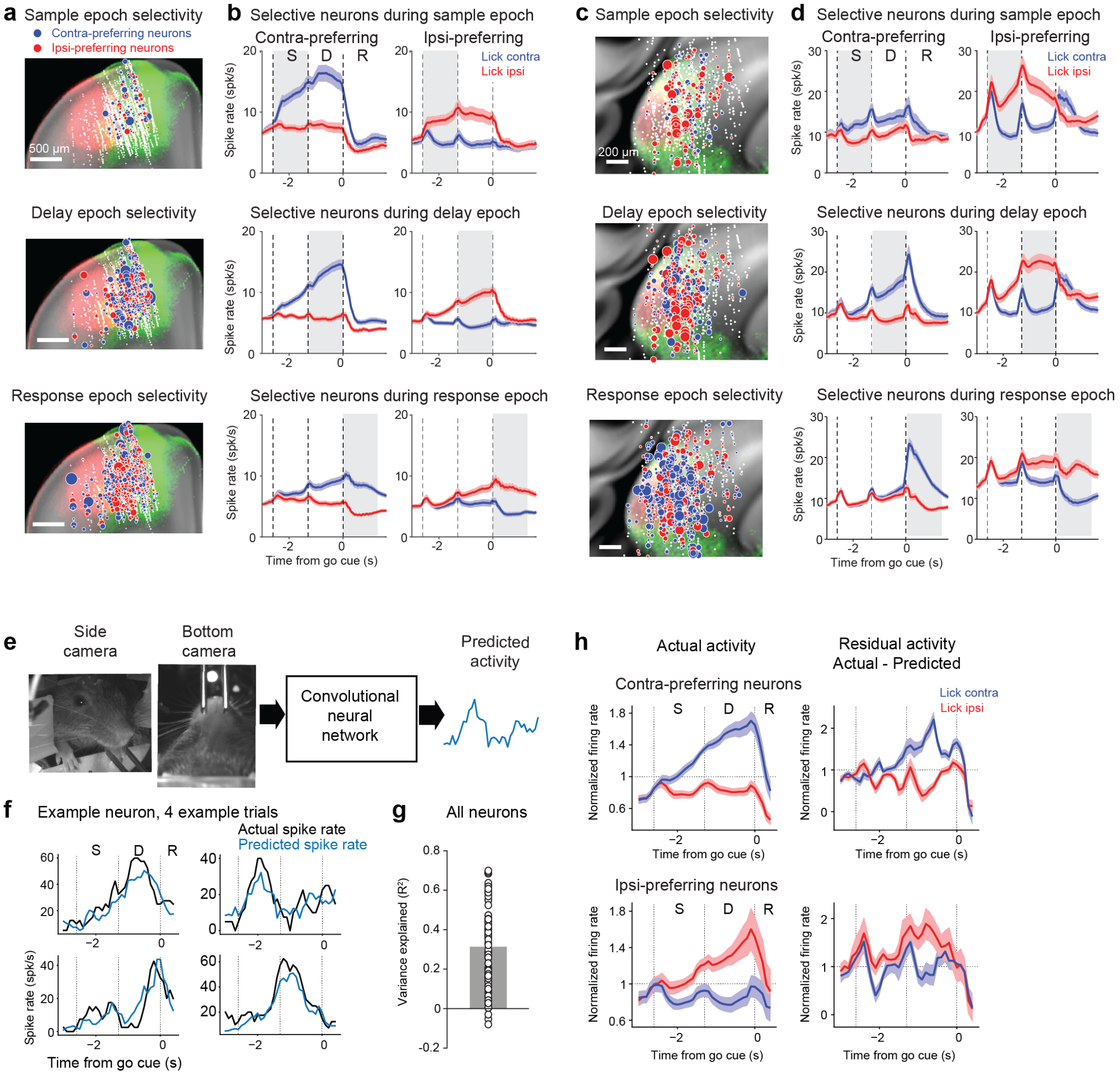

Supplementary Fig. 4 | Representation of contralateral and ipsilateral choices in ALM and SC.

- **a.** Spatial maps of contra-preferring (blue) and ipsi-preferring neurons (red) during specific epochs in CCF (2.90-3.91 mm posterior from bregma). Dot size indicates the size of the selectivity during specific epochs. Selectivity is the firing rate difference between preferred and non-preferred trial type. The neuronal selectivity maps are overlaid onto topographical ALM-SC descending projections. Fluorescence shows a medial ALM injection (green) and a lateral ALM injection (red). Data from Fig 2a replotted here.
- **b.** Population response of SC neurons with significant trial-type selectivity during specific epochs. *Top*, neurons with significant selectivity during the sample epoch (p<0.01, two-tailed t-test). *Middle*, neurons with significant selectivity during the delay epoch. *Bottom*, neurons with significant selectivity during the response epoch. For each epoch, neurons are sorted by their preferred trial type using spike counts from 10 trials and the remaining data was used to compute the population response (mean ± s.e.m. across neurons). Blue, response in lick contra trials; red, lick ipsi trials. Lick direction is relative to the recorded hemisphere.
- **c.** Same as **a** but for SC. Fluorescence shows ALM projections in SC from the medial (green) and lateral ALM (red) injections in **a**. Data from Fig 2a replotted here.
- **d.** Same as **b** but for SC neurons. Note rows 1-2, many SC neurons with ipsi-preferring selectivity during the sample and delay epochs switch their preference in the response epoch to become contra-preferring.
- **e.** We trained convolutional neural networks to predict single-trial activity of individual ALM and SC neurons from bottom- and side-view videos (Methods).
- **f.** An example ALM neuron activity (black) and predicted activity from videos (blue). 4 example trials. Dashed line, behavioral epochs.

g. Cross-validated R^2 of activity prediction. Bar, mean; circles, individual neurons (n = 92). Only neurons with significant selectivity during the delay epoch are included. ALM and SC neurons are combined due to the limited number of sessions with video data.

h. *Top*, response of contra-preferring ALM and SC neurons in lick contra (blue) and lick ipsi (red) trials. After subtracting the model-predicted activity, choice selectivity remains in the residual activity. Mean ± s.e.m. across neurons. *Bottom*, ipsi-preferring neurons.

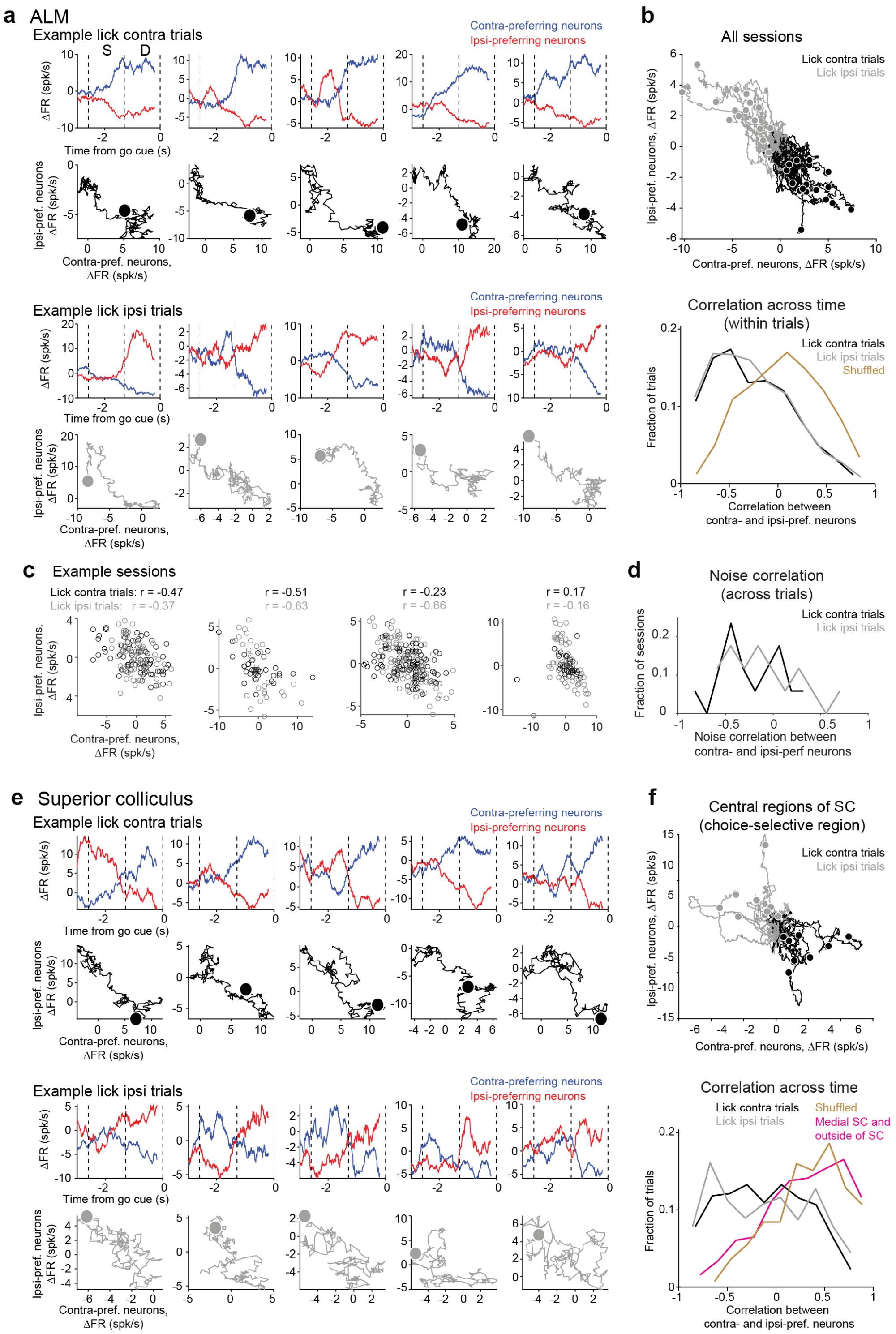

Supplementary Fig. 5 | Push-pull dynamics of contra-preferring and ipsi-preferring neurons.

- **a.** Activity (ΔFR) of contra-preferring and ipsi-preferring ALM neurons in several example trials. Data from an example session. Activity reflects firing rate change from the mean where the mean firing rate across trial types is subtracted to yield ΔFR (Methods). *Top rows*, ΔFR of contra-preferring (blue) and ipsi-preferring neurons (red) over time. Dashed line, behavioral epochs. *Bottom rows*, ΔFR of contra-preferring versus ipsi-preferring neurons in single trials. Dots show ΔFR at the end of the delay epoch.

- **b.** *Top*, average ΔFR of contra-preferring versus ipsi-preferring neurons in single sessions. Individual lines show individual sessions. Dots show ΔFR at the end of the delay epoch. Data from 17 sessions from 9 mice, 20-47 neurons per session. Only sessions with 5 or more selective neurons in each population simultaneously recorded for 30 or more trials are considered. *Bottom*, Pearson's correlation between ΔFR of contra-preferring and ipsi-preferring neurons in single trials. Correlation is calculated using ΔFR during the sample and delay epochs. Data from Fig. 2d replotted here for comparison. Data from 1606 trials. Black, lick contra trials; gray, lick ipsi trials; yellow, shuffled control, neurons are randomly grouped into two populations without regard to their choice preferences. Correlations are significantly negative compared to shuffled control, $p<0.001$, two-tailed t-test.
- **c.** 4 example sessions showing anticorrelated activity of contra- vs. ipsi-preferring neurons across trials. Individual dots show spike rates of contra- and ipsi-preferring populations in individual trials. Spike rate is calculated at the end of the delay epoch. Mean spike rate of each trial type is subtracted to yield ΔFR (Methods). Pearson's correlation (noise correlation) for each trial type is shown on top.
- **d.** Noise correlation across all sessions (n=17). Noise correlations are significantly negative compared to shuffled control, $p=0.0017$, two-tailed t-test.
- **e.** Same as **a** but for SC.
- **f.** Same as **b** but for SC. Recordings from the central region of SC where choice-selective neurons are enriched: 9 sessions from 5 mice, 13-30 neurons per session. Recordings that missed the central region of SC, including penetrations in the medial SC or outside of SC: 11 sessions from 7 mice, 11-25 neurons per session. Correlations in lick contra and lick ipsi trials are significantly negative compared to shuffled control, $p<0.001$, two-tailed t-test.

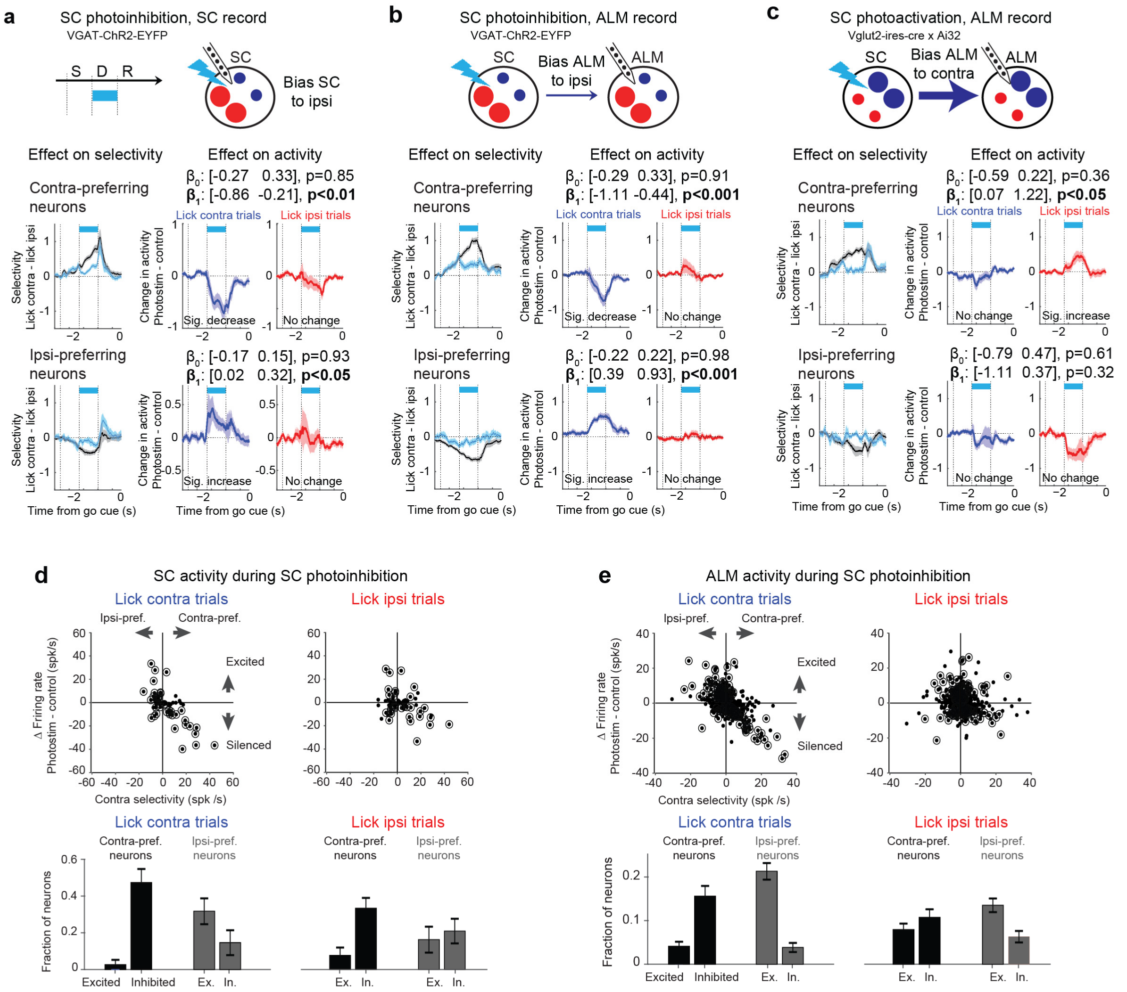

Supplementary Fig. 6 | SC manipulations induce trial-type-specific biasing of choice activity.

- **a.** Effect of SC photoinhibition on SC activity. *Left*, trial-type selectivity in control (black) and photoinhibition (cyan) trials. Selectivity is the firing rate difference between lick contra and lick ipsi trials. *Right*, activity change caused by photoinhibition in lick contra (blue) and lick ipsi (red) trials. Coefficients β_0 and β_1 are from a linear mixed effect model (Methods). 95% confidence intervals and p values show deviation from 0. Significant β_0 indicates non-specific activity change that occurs in all trial types. Significant β_1 indicates activity change that occurs only in one trial type. The activity change for the linear model is calculated using the spike rate during the last 200 ms of the delay epoch. SC photoinhibition decreases activity in contra-preferring neurons (i.e., negative β_1) but enhances activity in ipsi-preferring neurons (i.e., positive β_1). The activity changes occur only in lick contra trials (i.e., β_0 does not significantly differ from 0).
- **b.** Effect of SC photoinhibition on ALM activity. SC photoinhibition decreases activity in contra-preferring neurons but enhances activity in ipsi-preferring neurons. The activity changes occur only in lick contra trials.
- **c.** Effect of SC photoactivation on ALM activity. SC photoactivation enhances activity in contra-preferring neurons but decreases activity in ipsi-preferring neurons. The activity changes occur only in lick ipsi trials.
- **d.** Effect of SC photoinhibition on firing rate of SC neurons in VGAT-ChR2-EYFP mice. *Top*, firing rate change vs. strength of contra selectivity. Firing rate change is calculated on lick contra (left) and lick ipsi (right) trials separately. Dots, individual neurons. Circles, neurons with significant firing rate change during photostimulation ($p<0.01$, two-tailed t-test). *Bottom*, fraction of neuron excited or inhibited by the photoinhibition. Neurons are sorted by their choice preference.
- **e.** Effect of SC photoinhibition on firing rate of ALM neurons. In both ALM and SC, SC photoinhibition decreases activity in contra-preferring neurons but enhances activity in ipsi-preferring neurons. This effect is primarily induced in lick contra trials. In lick ipsi trials, SC photoinhibition induces a mixture of excitation and inhibition across individual neurons in both contra-preferring and ipsi-preferring populations.

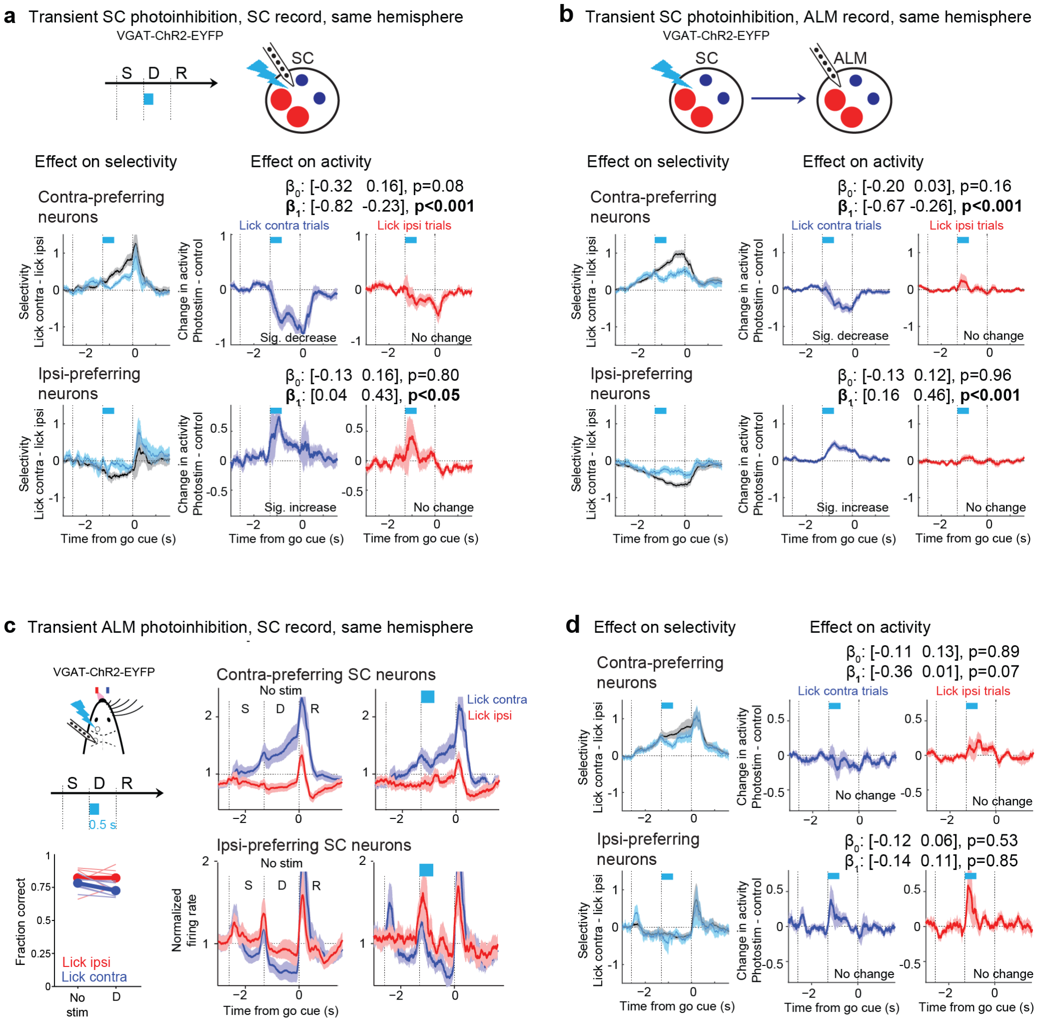

Supplementary Fig. 7 | Effects of transient unilateral SC photoinhibition and ALM photoinhibition on choice activity.

- **a.** Effect of transient unilateral SC photoinhibition on SC activity. *Left*, trial-type selectivity in control (black) and photoinhibition (cyan) trials. Selectivity is the firing rate difference between lick contra and lick ipsi trials. *Right*, activity change caused by photoinhibition in lick contra (blue) and lick ipsi (red) trials. Coefficients β_0 and β_1 are from a linear mixed effect model (Methods). 95% confidence intervals and p values show deviation from 0. Significant β_0 indicates non-specific activity change that occurs in all trial types. Significant β_1 indicates activity change that occurs only in one trial type. Significant activity changes are observed at the last 200 ms of the delay epoch, long after cessation of the photostimulus. SC photoinhibition decreases activity in contra-preferring neurons (i.e., negative β_1) but enhances activity in ipsi-preferring neurons (i.e., positive β_1). The activity changes occur only in lick contra trials (i.e., β_0 does not significantly differ from 0).
- **b.** Effect of transient unilateral SC photoinhibition on ALM activity. SC photoinhibition persistently decreases activity in contra-preferring neurons but enhances activity in ipsi-preferring neurons. The activity changes occur only in lick contra trials.
- **c.** Effect of transient unilateral ALM photoinhibition on behavioral choice and SC activity. *Left,* schematic of experimental manipulation and behavioral performance. Photostimulation is during the early delay epoch. N=6 mice. “Lick left” and “lick right” trials are grouped by instructed lick direction relative to the manipulated hemisphere. Blue, contralateral (lick contra); red, ipsilateral (lick ipsi). Photostimulation power, 1.2-1.5 mW. Right, comparison of activity in contra-preferring and ipsi-preferring SC neurons during control (*left*) and photostimulation (*right*). Only neurons with significant trial-type selectivity during the delay epoch are included. The spike rate of each neuron is normalized to the mean spike rate across all trial types. Mean ± s.e.m. across sessions (29 sessions, 6 mice).
- **d.** SC activity change caused by transient unilateral ALM photoinhibition in lick contra (blue) and lick ipsi (red) trials. Same as **a**. Activity at the last 200 ms of the delay epoch is not significantly altered relative to control trials as quantified by the linear mixed effect model.

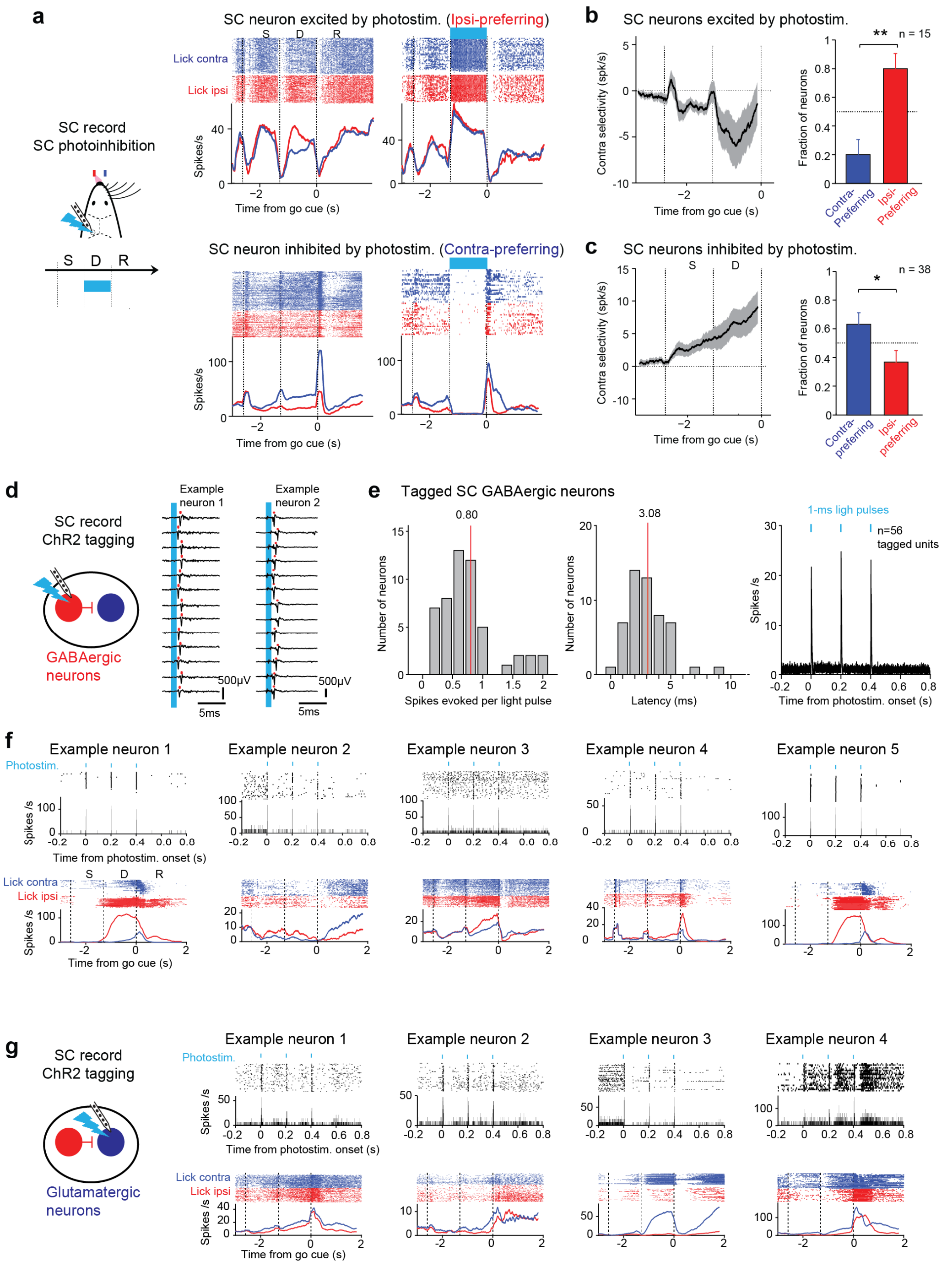

Supplementary Fig. 8 | ChR2 tagging of SC GABAergic and glutamatergic neurons.

- **a.** SC optrode recording during SC photostimulation in VGAT-ChR2-EYFP mice. *Top,* example ipsi-preferring SC neuron that is excited during photostimulation. Raster and PSTH for control (*left*) and photostimulation trials (*right*). *Bottom,* example contra-preferring SC neuron that is inhibited during photostimulation. Trial types are colored based on instructed lick direction relative to the recorded hemisphere (blue, lick contra trials; red, lick ipsi trials).
- **b.** SC neurons excited by photostimulation are predominantly ipsi-preferring. *Left*, contra-selectivity across the SC neurons excited during photostimulation (mean ± s.e.m.). Only neurons with significant trial-type selectivity during the delay epoch are included (n=15 from 7 mice). Contra-selectivity is the spike rate difference between lick contra and lick ipsi trials. *Right*, proportions of the excited neurons that are contra-preferring (blue) and ipsi-preferring (red). ** p<0.01, one-tailed test, bootstrap.

c. SC neurons inhibited by photostimulation are predominantly contra-preferring (n=38 from 7 mice). * $p<0.05$, significantly more contra-preferring neurons than ipsi-preferring neurons, one-tailed test, bootstrap.

d. ChR2-tagging of SC GABAergic neurons. Recording traces for two example neurons activated by 1-ms photostimulation of SC. Cyan, photostimulation. Red ticks, individual spikes. Neurons show short-latency response with high spike probability. These neurons are deemed GABAergic neurons (tagged).

e. *Left*, number of spikes evoked per light pulse in tagged GABAergic neurons. Photostimulus consists of 3 1-ms light pulses at 200 ms interval. Number of spikes evoked per light pulse is calculated as the average number of spikes in a 10-ms window following the light onset minus the average number of spikes in a 10-ms window prior to light onset. Mean, 0.8 spikes evoked per light pulse. *Middle*, spike latency of tagged GABAergic neurons. Mean, 3.08 ms. *Right*, average response of tagged GABAergic neurons to photostimulation.

f. Example tagged GABAergic neurons. *Top*, raster and PSTH aligned to photostimulus onset. Photostimulation was performed outside of the behavioral task. *Bottom*, raster and PSTH during the behavioral task. Trial types are colored based on instructed lick direction relative to the recorded hemisphere (blue, lick contra trials; red, lick ipsi trials).

g. ChR2-tagging of SC glutamatergic neurons and example tagged neurons.

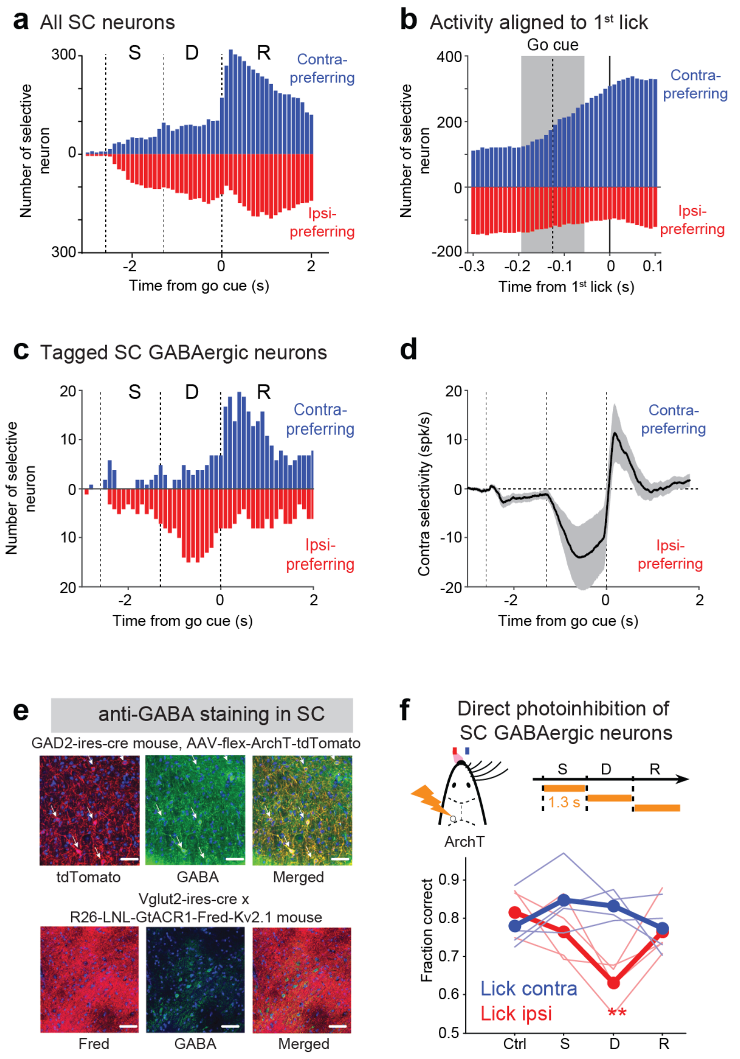

Supplementary Fig. 9 | Distinct selectivity of SC GABAergic neurons across epochs and direct photoinhibition of SC cell types.

- **a.** Equal proportions of SC neurons encode contralateral and ipsilateral choices during the delay epoch. Data from Fig. 2b replotted here for comparison. Number of significantly selective SC neurons as a function of time. Significant selectivity is based on spike counts in 200-ms time windows, $p<0.01$, two-tailed t-test. Neurons are sorted by their lick direction preference (blue, contra-preferring; red, ipsi-preferring). Dashed lines, behavioral epochs.
- **b.** SC neurons become contra-preferring immediately after the go cue. Number of significantly selective SC neurons as a function of time around movement initiation. Activity is aligned to the first lick. Dashed line and shade, onset time of the go cue, mean ± SD across trials.
- **c.** SC GABAergic neurons show preference for ipsilateral choice during the delay epoch but show preference for contralateral movement during the response epoch. Number of significantly selective SC GABAergic neurons at different time in the task. All tagged SC GABAergic neurons (n=29).
- **d.** Contra-selectivity across SC GABAergic neurons (mean ± s.e.m.). Note the reversal of selectivity after the go cue.
- **e.** Histology images showing ArchT and GtACR1 expression. *Top*, confocal images taken from the lateral SC of a GAD2-ires-cre mouse injected with AAV5-flex-ArchT-tdTomato virus. Arrows, all of the neurons expressing ArchT (red) are GABAergic (green). *Bottom*, confocal images taken from the lateral SC of a Vglut2-ires-cre x R26-LNL-GtACR1-Fred-Kv2.1 mouse. All of the GABAergic neurons (green) do not overlap with GtACR1 expression (red). Scale bars, 50 µm.
- **f.** Performance with direct photoinhibition of SC GABAergic neurons during the sample, delay, or response epoch. AAV-flexed-ArchT viruses in SC of GAD2-ires-cre mice. Direct photoinhibition of SC GABAergic neurons produces contralateral biases in lick direction primarily during the delay epoch. Thick lines, mean; thin lines, individual mice (n=8). $^{**}p<0.001$, one-tailed test, bootstrap (Methods). "Lick left" and "lick right" trials are grouped by instructed lick direction relative to the manipulated hemisphere. Blue, contralateral (lick contra); red, ipsilateral (lick ipsi). Photostimulation power, 10 mW.
